# Endoplasmic Reticulum Stress: A Critical Molecular Driver of Endothelial Dysfunction and Cardiovascular Disturbances Associated with Diabetes

**DOI:** 10.3390/ijms20071658

**Published:** 2019-04-03

**Authors:** Hatem Maamoun, Shahenda S. Abdelsalam, Asad Zeidan, Hesham M. Korashy, Abdelali Agouni

**Affiliations:** 1 Department of Medical Biochemistry and Molecular Biology, Faculty of Medicine, Ain Shams University, Abbaseyya, Cairo 11566, Egypt; hatem_maamoun@med.asu.edu.eg; 2 Department of Pharmaceutical Sciences, College of Pharmacy, QU health, Qatar University, P.O. Box 2713, Doha, Qatar; sa1512854@student.qu.edu.qa (S.S.A.); hkorashy@qu.edu.qa (H.M.K.); 3 Department of Basic Sciences, College of Medicine, QU health, Qatar University, P.O. Box 2713, Doha, Qatar; a.zeidan@qu.edu.qa

**Keywords:** endoplasmic reticulum stress, unfolded protein response, endothelial dysfunction, atherosclerosis, diabetes

## Abstract

Physical inactivity and sedentary lifestyle contribute to the widespread epidemic of obesity among both adults and children leading to rising cases of diabetes. Cardiovascular disease complications associated with obesity and diabetes are closely linked to insulin resistance and its complex implications on vascular cells particularly endothelial cells. Endoplasmic reticulum (ER) stress is activated following disruption in post-translational protein folding and maturation within the ER in metabolic conditions characterized by heavy demand on protein synthesis, such as obesity and diabetes. ER stress has gained much interest as a key bridging and converging molecular link between insulin resistance, oxidative stress, and endothelial cell dysfunction and, hence, represents an interesting drug target for diabetes and its cardiovascular complications. We reviewed here the role of ER stress in endothelial cell dysfunction, the primary step in the onset of atherosclerosis and cardiovascular disease. We specifically focused on the contribution of oxidative stress, insulin resistance, endothelial cell death, and cellular inflammation caused by ER stress in endothelial cell dysfunction and the process of atherogenesis.

## 1. Introduction

In recent decades, obesity has reached a global epidemic status among adults as well as pediatric populations. The high prevalence of obesity leads to dramatic negative consequences on morbidity, mortality, and quality of life in addition to a significant social and economic burdens. Altogether this has led to a reduced life expectancy among obese subjects [1,2]. Cardiovascular events associated with diabetes affecting coronary and peripheral arteries, enhance the risk of myocardial infarction and stroke which are the major root of premature death accounting for 80% of early mortality in diabetic patients [3].

Much evidence in the literature reported a strong association between obesity and cardiovascular diseases including acute coronary syndrome, hypertension, and heart failure [1,2]. Obesity is characterized by insulin resistance, a serious metabolic dysfunction, in addition to low grade inflammation and oxidative stress, which represent common risk factors for atherosclerosis. Enlarged adipose tissue secretes a variety of adipokines that play crucial roles in insulin resistance, coagulation, and inflammatory responses, thus, contributing to all the stages of atherogenesis from early endothelial dysfunction to lipid infiltration and plaque buildup [4]. Atherosclerosis precedes the development of coronary heart disease, and obese subjects were shown to exhibit larger vascular lesions compared to normal weight controls [5].

Obesity is also a major risk factor for type-2 diabetes which is associated with multiple cardiovascular disturbances [1,2]. Microvascular and macrovascular complications are pathological hallmarks of diabetes, resulting in severe damage and failure of several organ systems, mostly encompassing the eyes, nerves, kidneys, and the heart [6]. Sadly, diabetes is considered as the principal cause of blindness, non-traumatic lower limb amputation, and chronic nephropathy requiring dialysis or renal replacement in addition to ischemic vascular disorders [3]. In 2016, diabetes directly caused 1.6 million deaths worldwide [7], and the number of deaths has further risen to 4 million in 2017 [8]. Because of the alarming rates of morbidity and mortality implicated with diabetes, prevention and effective treatment strategies of complications are a pressing matter more than ever. 

Cardiovascular disease complications associated with obesity and diabetes are closely linked to insulin resistance and its complex implications on vascular cells particularly endothelial cells. Insulin induces its vascular effects mainly by enhancing the bioavailability of endothelium-derived nitric oxide (NO) through activating endothelial NO synthase (eNOS) [9]. Conditions of insulin resistance result in the impairment of endothelial-mediated vasodilation generally referred to as “endothelial dysfunction” [10]. Endothelial dysfunction is marked by impaired bioavailability of NO profoundly affecting peripheral vascular resistance in addition to the delivery of blood and nutrients to tissues involved in metabolic processes, thereby, contributing to metabolic disturbances. It is considered to be an independent prognosticator of cardiovascular events by playing a central role in the initiation of atherosclerosis and the progression of clinical complications consistently associated with diabetes.

It has been found that endoplasmic reticulum (ER) stress response is activated in insulin resistance states, such as obesity and diabetes, and contributes to insulin resistance and disruption of lipid metabolism [11,12,13] as well as cardiovascular disease [14,15]. The ER is the largest intracellular compartment where protein synthesis and post-translational maturation take place. If the balance between unfolded proteins and folding capacity of the ER is disturbed, a state of stress arises within the ER, leading to the bracing of a pro-survival pathway known as the unfolded protein response (UPR). The activation of UPR aims at reducing protein load on the ER and improving its homeostasis. However, the lengthened or chronic activation of UPR is recognized as a state of ER stress which is associated with the stimulation of pro-inflammatory and pro-apoptotic pathways [16,17]. 

In the current review article, we reviewed the role of ER stress in endothelial dysfunction, the crucial initiating step in atherogenesis sequence. We also highlighted the inter-connection between insulin resistance and impaired endothelial function in obesity and diabetes and the bridging role of ER stress in this process. 

## 2. Physiological Roles of Endothelium and Endothelial Dysfunction

### 2.1. Physiological Roles of Endothelium

For many years, the endothelium was regarded only as a static biological barrier that wraps the innermost layer of all vessels of the vascular tree. However, extensive investigations shed light on the dynamic functions and paramount role of the endothelium in maintaining cardiovascular homeostasis. Now, the endothelium is recognized as a large functional endocrine organ possessing diverse secretory, metabolic, and immunological roles rather than just an inert lining. The endothelium is a single layer of tightly interconnected cells that line the vessel walls, starting from the heart to the narrowest capillaries. It consists of around $10^{13}$ cells spreading over a surface area of 4000 m^2^ in an adult. Endothelial cells are vital for orchestrating the flow of molecules, nutrients, and blood cells and their functions are fine-tuned through the interaction of several metabolites, proteins, and hormones with their endothelial membrane-bound receptors [18,19,20,21,22]. 

The most crucial endothelium-derived factor is NO, which is known to mediate important roles, such as maintaining normal vascular homeostasis and an anti-thrombotic vascular surface. NO is produced by eNOS and then diffuses into the adjacent smooth muscle where it activates guanylyl cyclase which generates cyclic guanosine monophosphate (cGMP) that then activates a cGMP-dependent protein kinase responsible for the phosphorylation of myosin light chain (MLC) phosphatase. MLC phosphatase will then dephosphorylate MLC leading eventually to smooth muscle relaxation [21,22]. Furthermore, NO can prevent the aggregation of platelets and growth of smooth muscle cells in addition to blocking mRNA transcription of several adhesion molecules, such as vascular cell adhesion molecule (VCAM)-1 and intercellular adhesion molecule (ICAM)-1 [23]. The force exercised by shear stress on the surface of vascular walls and the interaction of a variety of agonists with their surface receptors (e.g., acetylcholine and bradykinin) can stimulate eNOS causing the synthesis and release of NO from endothelial cells. Other major vasoactive factors secreted by the endothelium include endothelium-derived hyperpolarizing factor (EDHF) and prostacyclin (PGI2), which contribute to the vasodilatory effect in addition to platelet inhibition by PGI2 [21,22,24].

Endothelial cells also secrete a strong vasoconstrictor factor, endothelin-1 (ET-1), that possesses both inflammatory and proliferative effects and, hence, contributes to the pathogenic processes in the cardiovascular system. ET-1 antagonizes the effects of NO at multiple molecular levels. It directly inhibits eNOS and, hence, reduces NO release, increases reactive oxygen species (ROS) production by activating Nicotinamide Adenine Dinucleotide Phosphate (NADPH) oxidase, responsible for the production of superoxide anion (O_2_^−^), which can scavenge NO to form peroxynitrite (ONOO^−^), a potent free radical contributing further to the reduction of NO bioavailability. In addition, ET-1 blocks tetrahydrobiopterin (BH_4_) resulting in the uncoupling of eNOS which starts producing O_2_^−^ instead of NO [25,26]. The endothelium also generates several other vasoconstrictor agents including prostaglandin H2 (PGH2), thromboxane A2 (TXA2), and ROS. Furthermore, endothelial cells express at their surface angiotensin-converting enzyme (ACE) which controls the conversion of angiotensin I (angI) into angiotensin II (angII), another very potent vasoconstrictor and mitogenic molecule [27,28,29]. NO release by endothelial cells contributes to preventing leukocyte adhesion in normal conditions. However, activated endothelium expresses multiple adhesion molecules at its surface, which facilitates the recruitment and aids the passage of monocytes across the vessel wall and contributes, thus, to the promotion of an inflammatory micro-environment. Activated endothelial cells can produce and release several pro-inflammatory cytokines (e.g., tumor necrosis factor (TNF)-α and interleukin (IL)-6) [30]. 

The endothelium also controls angiogenesis, which is stimulated by certain pro-angiogenic molecules, particularly vascular endothelial growth factor (VEGF), platelet activating factor (PAF), and fibroblast growth factor (FGF). VEGF-A is a central factor regulating the formation of blood vessels; it is widely expressed in tissues frequently making new vessels, such as diabetic retina, cardiac tissue, and the endothelium [31]. By interacting with its receptor expressed by endothelial cells, VEGF activates the mitogen-activated protein kinase (MAPK) cellular pathway, which then promotes angiogenesis through the transactivation of key genes implicated in this process. However, this process can be counterbalanced by the release, by endothelial cells, of other anti-angiogenic factors, such as angiostatin and thrombospondin, which can block the angiogenic response [21,22,32].

### 2.2. Endothelial Dysfunction

The state of the endothelium is referred to as dysfunctional when it is no longer able to maintain its natural function and switches towards one of the following states: (I) reduced bioavailability of NO as a consequence of reduced production and/or scavenging by excessive ROS release; (II) weakened endothelium-mediated vasodilation; (III) disrupted fibrinolysis and enhanced thrombosis; (IV) overproduction of growth factors, chemokines, adhesion molecules, pro-inflammatory molecules (e.g., cytokines), ROS, and altered (impaired or excessive) angiogenesis. Because of its crucial contributing role to the main functions of endothelial cells, reduced production and liberation or NO and/or reduced activation of eNOS are the earliest and most significant features of endothelial dysfunction. Endothelial dysfunction is the early step in the advancement of cardiovascular diseases by playing a central role in the initiation of atherosclerosis and progression of clinical complications consistently associated with diabetes [33,34,35].

## 3. Unfolded Protein Response (UPR) and Endoplasmic Reticulum (ER) Stress

### 3.1. The Physiological Roles of ER

Being the largest organelle, the ER is a highly active multifunctional entity with a paramount role in cell homeostasis, functioning, and survival. Two types of ER exist inside the cell; the rough and smooth ER. The ribosomes enclosed at the surface of the rough ER mediate protein synthesis, folding, and post-translational modifications. Smooth ER, however, does not express ribosomes and is, thus, not capable of protein synthesis, but it plays a key role in lipid and steroid synthesis, carbohydrate metabolism, and intracellular calcium handling [36]. Translation of proteins occurs in the cytosolic surface of the rough ER via ribosomes. After translation, proteins are transferred inside the lumen of the ER where they are subjected to post-translational modifications, folding, and stabilization into their functional structure. These functions are performed by the ER folding machinery, composed of foldases and molecular chaperones, such as the immune-globulin protein (BiP) also known as Glucose regulated protein 78 (GRP78), which are dedicated to folding proteins into their proper form and prevent their aggregation. Moreover, they also act as a quality control checkpoint, assessing the state of newly synthesized proteins. The nascent polypeptide chain is partially folded inside the ER before being exported to the Golgi apparatus for further post-translational maturation to gain the final conformation [36]. However, if proteins are misfolded or aggregated, they are retained within the ER where they enter the ER discarding system known as ER-associated degradation (ERAD) machinery. The ERAD system degrades aggregated and misfolded proteins with the help of the proteasome system, thus, preventing them from exiting the ER and causing harm to the cellular environment [37,38].

### 3.2. UPR and ER Stress Response

**Figure 1:**
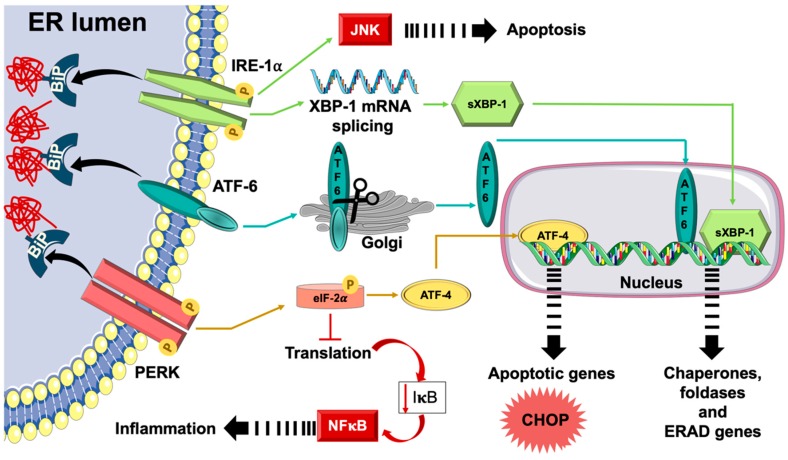
Unfolded protein response (UPR). Upon the buildup of misfolded and/or unfolded proteins inside the endoplasmic reticulum (ER), the chaperone, binding immunoglobulin protein (BiP), dissociates from and, thus, activating the three ER transmembrane proteins, protein kinase-like endoplasmic reticulum kinase (PERK), activating transcription factor (ATF)-6, and inositol requiring enzyme (IRE)-1α. Activated PERK phosphorylates eukaryotic initiation factor (eIF)-2α blunting, thus, general translation of proteins with the selective synthesis of transcription factor, ATF-4, being allowed. Free ATF-6 transfers to the Golgi apparatus where it gets cleaved and, hence, activated. Activated cleaved ATF-6 relocates to the nucleus and boosts the transcription of molecular chaperones that can help in improving ER protein folding functions. Activated IRE-1α causes the mRNA splicing of X-box binding protein (XBP)-1 and generates an active splice variant (sXBP-1) which stimulates the transcription of genes controlling the expression of several molecular chaperones, foldases, and proteins the ER-associated degradation (ERAD) machinery aiming at restoring ER homeostasis. Prolonged activation of UPR leads to ER stress with activation of inflammatory signaling pathways, including c-Jun N-terminal Kinase (JNK) and nuclear factor (NF)-κB, which may activate pro-inflammatory and apoptotic signals, such as CCAAT/enhancer-binding protein homologous protein (CHOP).

As highlighted above, the ER is a complex organelle with various functions, and it is kept under tight regulation to maintain a balanced and adequate function. Despite this regulation and the presence of numerous chaperones and foldases, unfolded and misfolded proteins may accumulate inside the ER lumen. This occurs when the demand for folding newly synthesized peptides outpaces the folding capacity of the ER, such as in cases of metabolic disturbances (e.g., obesity and diabetes). Such perturbations overwhelm the ER leading to the activation of the unfolded protein response (UPR) pathway (Figure 1). The UPR initial aim is to recover ER homeostasis by I) ceasing the translation of protein to prevent further load, II) increasing the ER folding capacity, and III) activating the ERAD system to dispose of the unrepairable misfolded proteins. Yet, when these attempts fail to re-establish normal ER functions and alleviate the stress, cellular inflammation and apoptotic signals are triggered [37,39,40,41].

The UPR operates through the activity of three main ER-bound sensors; protein kinase-like endoplasmic reticulum kinase (PERK), activating transcription factor (ATF)-6, and inositol requiring enzyme (IRE)-1α. These effectors have two domains; C-termini found inside the ER and N-termini in the cytosol. Under normal non-stress state, the luminal domains of the three effectors interact with the chaperone BiP. BiP keeps the enzymatic activities of these effectors in inactive states. Nonetheless, the buildup of unfolded and/or misfolded proteins calls upon BiP resulting in its detachment from the UPR sensors and, hence, activating them (Figure 1) [37,39,40,41].

PERK is the first protein in the UPR pathway [13,42]. PERK dimerizes and autophosphorylates after its dissociation from BiP, then it phosphorylates eukaryotic initiation factor (eIF)-2α. Phosphorylated eIF-2α attenuates mRNA translation to reduce the load on the ER; however, it selectively enhances the mRNA translation of ATF-4. ATF-4 promotes the transcription of genes encoding for amino acid synthesis and ER stress-mediated apoptosis; such as tribbles homolog 3 (TRIB3) and CCAAT/enhancer-binding protein homologous protein (CHOP), respectively (Figure 1). The negative feedback of PERK signaling is mediated by ATF-4 which enhances the transcription of growth arrest and DNA damage inducible protein (GADD34) that leads to PERK dephosphorylation and inactivation by protein phosphatase 1 (PP1) [37,39,40,41].

The second UPR sensor is ATF-6 [13,42], which dissociates from BiP due to the increase in unfolded and/or misfolded proteins and then translocates to the Golgi body for further processing and cleavage by Site 1 protease (S1P), a serine protease, and site 2 protease (S2P), a metalloprotease that cleaves the N-terminal cytosolic domain. Cleaved ATF-6 translocates then to the nucleus and induces the expression of multiple ER stress response genes especially molecular chaperones, such as BiP and glucose regulated protein 94 (GRP94), to further assist in the correcting protein folding. If the UPR response fails to resolve the stress condition and the state of stress pursues in chronic mode, the cell may finally decide to undergo apoptosis where ATF-6 will play a critical role through the induction of CHOP expression (Figure 1) [43,44]. It is noteworthy that the exact time point at which the cell reveres from a pro-survival mode to an apoptotic one remains elusive.

The last key effector in the UPR response is IRE-1α which features both a protein kinase function and an endo-ribonuclease activity. When BiP dissociates freeing it up from its inhibition, it undergoes oligomerization resulting in auto-phosphorylation and subsequent stimulation of its endo-ribonuclease activity which splices an intron in the mRNA sequence of the X-box binding protein (XBP)-1, leading to the expression of the active splice variant of XBP-1. Spliced XBP-1 stimulates the transcription of several genes encoding for molecular chaperones and proteins of the ERAD machinery to restore ER homeostasis (Figure 1) [45]. When the UPR fails to resolve this situation of stress, this same enzyme, IRE-1α, triggers an apoptotic signaling cascade by recruiting TNF-α receptor associated factor (TRAF)-2 and apoptosis signaling kinase-1 (ASK)-1 causing the phosphorylation and, hence, activation of inflammatory signaling molecules, p38 MAPK and c-Jun N-terminal Kinase (JNK), [46]. Activated JNK translocates then to the mitochondrial membrane and catalyzes the phosphorylation of the anti-apoptotic protein, Bcl-2, and pro-apoptotic protein, Bim, with subsequent inhibition and activation, respectively, resulting in the initiation of apoptosis [47]. Activated p38 MAPK phosphorylates and, hence, activates CHOP, which alters gene expression to favor apoptosis by the induction of Bim and suppression of Bcl-2, eventually stimulating caspases and apoptosis [48].

## 4. Contribution of ER Stress Response to Cardiovascular Disease

### 4.1. The Role of ER Stress in Endothelial Dysfunction

Sustained ER stress activation has been linked to several diseases including diabetes, cardiovascular disorders, and endothelial dysfunction [34]. Endothelial cells isolated from arteries susceptible to atherosclerosis revealed chronic stimulation of ER stress illustrated by the activation of UPR signal transducers specifically, IRE-1α and ATF-6 [49]. The expression of XBP-1 was found to be elevated in endothelial cells isolated from atherosclerotic lesions and curvatures of the aorta, while only a small amount was present in normal aorta. Furthermore, human umbilical vein endothelial cells (HUVECs) exposed to disrupted flow rather than laminar flow showed an increase in the expression of XBP-1. When *XPB-1* was over-expressed in HUVECs, an increase in the amount of spliced XBP-1 caused endothelial cell apoptosis through downregulating protein levels of VE-cadherin, a critical junctional protein, and the activation of caspases cascade. Intriguingly, it was shown that the transient activation of XBP-1 had an important role in endothelial cell proliferation, as proliferating HUVECs displayed an elevated expression of XBP-1 [50]. On the other hand, more recently, it has been reported that the exposure of HUVECs to a laminar flow inhibited ER stress and UPR activation, and prevented ER stress-mediated endothelial cell apoptosis [51]. Recent investigations of the specific role of XBP-1 in endothelial cell proliferation through a scratch migration assay to mimic wound healing, revealed that XBP-1 is crucial for activating endothelial cell migration and upregulating the expression of eNOS [52]. These findings suggest that the endothelial effects of XBP-1 on proliferation maybe mediated through the UPR of the ER (UPR^ER^) instead of ER stress response. In certain conditions, the cell activates UPR^ER^ as a pre-emptive protective mechanism when anticipating a surge in protein demand and synthesis to prevent the activation of ER stress. This response is thought to be mediated by the cytoprotective effects of XBP-1 which was shown to control stem cell differentiation in the absence of ER stress response [53].

ER stress induction in endothelial cells was shown to result in a significant upregulation of both mRNA and protein expression of ET-1, while diminishing those of eNOS [54]. Kassan et al. [15] have demonstrated that ER stress inhibition protected mice against angII-induced endothelial dysfunction, where mice treated with angII and ER stress inhibitor showed improved eNOS activity and enhanced phosphorylation in addition to improved endothelium-dependent vascular relaxation [15]. 

In healthy endothelium, maintaining vascular homeostasis and tone is a fine-tuned harmony between endothelium-derived relaxing factors (EDRF) and constricting factors (EDCF). In pathological conditions that disrupt endothelial function, perturbations of this balance occur resulting in conditions, such as hypertension. In a model of hypertensive rats, the administration of ER stress inhibitors (chemical chaperones); tauroursodeoxycholic acid (TUDCA) or 4-phenylbutyric acid (PBA), resulted in normalized vascular tone through suppressing cytosolic phospholipases A2 (cPLA2)/cyclooxygenases (COX) pathway and, hence, restoring the balance between EDRF and EDCF [55]. 

To further link ER stress to endothelial dysfunction, ER stress was induced in coronary artery endothelial cells by treating the cells with tunicamycin, an ER pharmacological stressor [48]. The induction of ER stress caused a reduction in the activity of eNOS as well as its expression in coronary endothelial cells. In addition, ER stress caused an increase in the phosphorylation of p38 MAPK which is known to antagonize the phosphorylation and, hence, activation of eNOS. Moreover, tunicamycin induced oxidative stress through the upregulation of mRNA expression of NADPH oxidase, the major source of ROS in the vasculature, which in turn impaired endothelial function by reducing NO bioavailability [48]. All of these effects were not observed in endothelial cells treated with tunicamycin in the presence of the chemical chaperone, TUDCA, to alleviate ER stress [56]. Similarly, Walsh et al. [57] demonstrated that endothelial dysfunction induced by hyperglycemia was alleviated by the administration of TUDCA indicating that ER stress is a mediator of hyperglycemic actions on endothelial cells [57]. More recently, HUVECs grown in intermittent high glucose environment for a duration of 5 days exhibited activated ER stress response evident by protein overexpression of BiP, ATF-4, and p-eIF-2-α (Ser51) compared to control conditions. Moreover, intermittent high glucose caused endothelial dysfunction in HUVECs by reducing NO production, increasing endothelial cell apoptosis and impairing angiogenic capacity. All these endothelial dysfunction features were prevented in the presence of chemical chaperone, PBA [58]. Prolonged administration of PBA was reported to attenuate endothelial dysfunction in streptozotocin (STZ)-induced diabetic rats [59]. Choi et al. [60] showed that *db/db* diabetic mice had a significant increase in protein expression of ER stress markers; BiP, p-IRE-1α and its downstream XBP-1 in addition to p-PERK and its downstream p-elF-2α, compared to non-diabetic mice. In addition, endothelial function was altered through impaired endothelium-dependent relaxation in mouse coronary arteries with a significant elevation in the expression of ICAM-1 and VCAM-1. The administration of the chemical chaperone, TUDCA, reversed this endothelial dysfunction in db/db mice [60]. Altogether, there is substantial evidence to support the crucial role of ER stress in unbalancing endothelial functions that pave the way for the onset of endothelial dysfunction associated with metabolic perturbations.

### 4.2. The Role of ER Stress in the Development of Atherosclerosis

In the atherogenic sequence, oxidative stress and sub-intimal retention of lipoproteins are the major triggers for the activation of endothelial cells and subsequent surface exposure of adhesion molecules, and release of cytokines, and chemokines, leading eventually to the enrollment of leukocytes, particularly monocytes. Monocytes differentiate in the sub-intimal layer into macrophages which can phagocyte oxidized lipoproteins, forming the famous foam cells that can undergo apoptosis and necrosis. ER stress has been found to contribute to most of these cellular disturbances, chiefly endothelial dysfunction, and, therefore, deciphering the molecular mechanisms underpinning the role of ER stress in atherosclerosis is of paramount importance in identifying novel therapeutic targets and approaches [61]. ER stress is a molecular link that can participate in the onset of diabetes-related atherosclerosis, especially in the late stages [62]. UPR has been found to be activated at all steps of atherosclerosis development [63].

Investigations in humans and rodent models of atherosclerosis have demonstrated the activation of ER stress response in both endothelial cells and macrophages. Free cholesterol and 7-ketocholesterol activated ER stress in macrophages and led to their cell death in a process involving CHOP; however, the deletion or silencing of CHOP in in vitro and in vivo models reduced apoptosis in macrophages and reduced the size and vulnerability of atherosclerotic plaques [64]. It has been observed that the IRE-1α/ASK-1/JNK axis strongly contributes to CHOP-induced macrophage cell death. For example, when apolipoprotein E (ApoE), which is essential for clearing triglycerides and cholesterol from endothelial cells and macrophages, was knockout in mice that were fed a western cholesterol-rich diet, mice exhibited macrophage and endothelial cell death that was reversed with small interfering RNA against IRE-1α or with a JNK inhibitor, harnessing, therefore, the role of ER stress signaling pathway in the progression of atherosclerotic lesions. The implication of ER stress in atherogenesis was further confirmed in studies that utilized chemical chaperones to improve ER homeostasis. In a genetic model of atherosclerosis, the administration of a chemical chaperone, PBA, to ApoE^−/−^ mice reduced plaque size and macrophage cell death [65]. More recently, the role of IRE-1α, the most conserved ER-bound effector, in the development of atherosclerosis was further harnessed by work conducted by Tufanli et al. [66] using an IRE-1α inhibitor (STF-083010). Authors reported that the treatment of macrophages with STF-083010 prevented ER stress-induced upregulation of several atherogenic genes, such as cytokines and chemokines, and blunted lipid-induced mitochondrial ROS production. In addition, the inhibition of IRE-1α reduced plaque area and increased its content in collagen, which confers elasticity to the plaques, in ApoE^−/−^ mice fed a western diet [66]. 

Aggravated atherosclerosis is a very common feature of diabetes and is intimately linked to hyperglycemia and dyslipidemia [34,67]. A major contributor to vascular complications mediated by hyperglycemia is through the non-enzymatic synthesis of advanced glycation end products (AGEs), which were reported to enhance the oxidization of low-density lipoprotein (LDL) to form oxidized LDL (ox-LDL) [34]. The treatment of macrophages with ox-LDL was reported to cause the activation of ER stress in a dose-dependent fashion and increased the expression of transporters that facilitate lipid uptake and metabolism, such as cluster of differentiation (CD)36. These effects were prevented by the chemical chaperone, TUDCA, and the silencing of CHOP [68]. Very recently Tian et al. [69] reported that glycated high-density lipoprotein (HDL) also contributes to atherogenesis via the activation of the ER stress response. Authors exposed RAW264.7 macrophages to glycated HDL, prepared from native HDL collected from healthy donors and then incubated with high glucose. Glycated HDL caused apoptosis of macrophages via the activation of ER stress, which was prevented in the presence of the chemical chaperone, PBA, or when CHOP or PERK were silenced. Similar findings were replicated using glycated HDL collected from diabetic patients. A focal role for autophagy in the effects mediated by glycated HDL was highlighted both in vitro and in vivo using an ApoE^−/−^ mouse model of atherosclerosis and an inhibitor of autophagy [69]. Work by Li et al. [70] has shed light on the role of apolipoprotein C3 (ApoC3), which is found in lipoproteins rich in triglycerides, in atherosclerosis. Transgenic mice overexpressing ApoC3 had high plasma triglyceride levels and exhibited neointimal hyperplasia compared to controls and ApoC3^−/−^ mice. Furthermore, transgenic ApoC3 mice were crossed with mice deficient for LDL receptor (LDLR^−/−^) resulted in accelerated atherosclerotic lesions [70]. Later, it was found that triglyceride-rich lipoproteins collected from transgenic ApoC3 mice caused the activation of ER stress response in a dose-dependent fashion in both HUVECs and peritoneal mouse macrophages. Furthermore, activated ER stress response was observed in aortas from ApoC3 transgenic mice crossed with LDLR^−/−^ mice [71]. 

These studies underscore the crucial role of ER stress as a converging molecular link in the progression of atherosclerosis through several actions, such as the activation of pro-apoptotic caspases and apoptosis of macrophages and vascular endothelial cells [58,72]. The induction of ER stress may also participate to impaired endothelial function and atherogenesis through insulin resistance [11,73,74] and lipid buildup in tissues by enhancing the expression and action of sterol regulatory element binding proteins (SREBP), that regulate lipid biosynthesis and uptake to endothelial cells [75].

## 5. Major Mechanisms Underpinning ER Stress-Mediated Endothelial Dysfunction

Several mechanisms have linked ER stress activation to endothelial dysfunction; however, the major mechanisms include insulin resistance, which has been linked to impaired NO production and signaling, oxidative stress through a reduction in NO bioavailability, endothelial cell apoptosis, and, finally, cellular inflammation and the activation of pro-inflammatory and anti-survival signaling hubs [34]. 

### 5.1. ER Stress-Mediated Insulin Resistance

Insulin plays critical roles in the metabolism of glucose and lipids, cell survival and growth as well as vascular functions among other important actions [76]. It exerts its action through the interaction with its receptor which undergoes auto-phosphorylation on tyrosine residues, thus, triggering the signaling cascade. Insulin signaling is then mediated through two major pathways; the phosphatidylinositol-3-kinase (PI-3K)/protein kinase B (PKB) pathway, which controls the majority of insulin’s metabolic actions, such as glucose uptake and anti-apoptotic actions, and the MAPK pathway, which along with PI-3K govern cell growth and differentiation [76]. 

A hallmark of metabolic disturbances occurring in diabetes is insulin resistance [77]. The impairment in the sensitivity and circulating levels of insulin profoundly affects endothelial function and contributes largely to the development and progression of diabetic micro- and macrovascular complications [78]. Okon et al. [79] showed that arteries from human diabetic patients had a 50% reduction in phosphorylated PKB levels in comparison to those of healthy subjects. These findings were accompanied by a perturbation of endothelium mediated-vasodilation, which correlated with decreased NO production as well as lower eNOS expression and activity [79]. On the other hand, to demonstrate the effect of insulin resistance on endothelial function, Duncan et al. [80] generated transgenic mice that over-express endothelial-specific mutant insulin receptor. These mice had severe endothelial dysfunction and diminished NO production [80]. 

ER stress is a common molecular feature in obesity and diabetes that are associated with insulin resistance. Several studies linked ER stress to impaired insulin signaling [77]. Özcan et al. [11] demonstrated that ER stress markers were elevated in high fat diet (HFD)-fed and genetically obese mice. Furthermore, it was shown that the induction of ER stress resulted in insulin resistance by weakening insulin transduction signal and preventing the phosphorylation of PKB [11]. In another investigation, it was also shown that ER stress induced insulin resistance through hindering insulin-stimulated glucose uptake [73]. Two main cellular mechanisms were proposed to link ER stress response to insulin resistance. The first mechanism is through the activation of IRE-1α branch of UPR pathway, which leads to the activation of JNK. Activated JNK elicits serine phosphorylation of insulin receptor substrate (IRS)-1, emanating in its inhibition and, hence, the impairment of the insulin signaling cascade [74]. The second mechanism is through the other UPR branch, PERK pathway. ATF-4 activates the transcriptional upregulation of tribbles-like protein 3 (TRB3), a PKB inhibitory kinase, that ultimately inhibits PKB and causes impairment of insulin signaling response [81]. Furthermore, ER stress activation may contribute to the impairment of insulin signaling through the accumulation of misfolded pro-insulin and suppression of protein translation of PKB, thereby, blunting the release of insulin as well as its signaling pathway. It has been observed that heterozygous mice expressing non-phosphorylable eIF-2α (Ser51 substituted by an alanine) developed diabetes when fed an HFD and had severely impaired insulin secretion and a defective trafficking of pro-insulin which accumulated within the ER lumen [82]. The pharmacological activation of ER stress in human choriocarcinoma JEG-3 cells caused an exaggerated apoptotic response through a mechanism involving reduced protein translation of PKB rather than an impaired phosphorylation. The effects of ER stress on JEG-3 cells were reversed by the overexpression of full-length (wild-type) PKB, but not kinase-defective PKB, indicating the role of ER stress in the translational downregulation of PKB [83].

In obesity, characterized by insulin resistance, ER stress activation was found to be associated with feto-placental endothelial dysfunction at birth in obese pregnant women [84]. Moreover, plasma from obese children caused insulin resistance by impairing insulin-stimulated NO production, a characteristic of endothelial dysfunction, in HUVECs with concomitant activation of ER stress response. The administration of ER stress chemical chaperones, PBA or TUDCA, reversed endothelial dysfunction in HUVECs [85]. In obese adults with known endothelial dysfunction, all UPR effectors, PERK, IRE-1α, and ATF-6, were activated as measured by quantitative immunofluorescence in antecubital veins [86]. Overall, these findings support that ER stress is involved in endothelial dysfunction induced by obesity and is intimately associated with insulin resistance. 

ER stress was also found to mediate HFD-induced impairment of insulin-controlled vasodilation which improved by the treatment of animals with TUDCA [87]. Recently, HFD-induced obesity was shown to cause ER stress and endothelial dysfunction, where there was an upregulation of the expression of ER stress markers accompanied with a rise in the expression of vascular endothelial adhesion molecules and a decrease in the expression of endothelial junction proteins. The alleviation of ER stress by the treatment of animals with PBA resulted in the protection of endothelial cells from HFD-induced perturbations and restored normal expression of endothelial adhesion proteins [88]. Furthermore, Agouni et al. [89] reported that the selective deletion of protein tyrosine phosphatase (PTP)-1B, a protein located on the ER membrane and a negative regulator for insulin receptor, in the liver protected mice from HFD-induced endothelial dysfunction in the aorta through an improvement of ER stress response [89]. Because of its key location at the ER membrane, PTP1B was found to crosstalk with ER stress [90,91].

### 5.2. ER Stress-Mediated Oxidative Stress

ER stress is intimately associated, in a vicious circle, with oxidative stress, a major contributor to endothelial dysfunction [34]. The cisternal space of the ER is highly oxidizing compared to the cytosolic compartment because of the high level of reduced glutathione (GSH) to encourage the establishment of native disulfide bonds between nascent protein chains [92]. When protein folding takes place inside the ER, the formation of disulfide bonds requires the oxidization of newly translated proteins under the action of isomerases. Errors in the proper pairing of cysteine residues between polypeptide chains generates non-native disulfide bonds, thus, leading to the accumulation of misfolded proteins. During the normal folding process, electrons are transferred through cysteine molecules in the nascent polypeptide chains. This transfer of electrons from chains to oxygen to form hydrogen peroxide (H_2_O_2_) is coordinated by the action of specialized isomerases, disulfide isomerase (PDI) and ER oxidoreductase (ERO)-1α. In conditions of high demand on protein synthesis, such as obesity and diabetes, an overwhelming formation of non-native disulfide bonds leads to overconsumption of GSH to scavenge ROS and protect the cell. The depletion of GSH stores in the cell leads to an increase in oxidative stress [34,92]. ROS were found to inactivate ER isomerases contributing, therefore, to the pileup of unfolded and misfolded proteins [93]. Protein folding requires adenosine triphosphate (ATP) and, hence, the buildup of misfolded or unfolded proteins stimulates glucose use and activates oxidative phosphorylation in mitochondria to generate more ATP with the subsequent generation of elevated amounts of ROS. 

The ER and mitochondria are both physically and physiologically interconnected intracellular organelles. During, the pro-survival phase of UPR, the relationship between the ER and mitochondria stimulates ATP synthesis through the shuttling of Ca^2+^ from the ER through to the mitochondria via specific structures called mitochondria-associated membranes (MAMs). Under ER stress conditions, enhanced demand for energy was shown to control the composition and functions of MAMs [94]. PERK-deficient cells were found to have lower MAMs, and to be associated with disturbed shuttling of Ca^2+^ from the ER to the mitochondria and reduced susceptibility to ROS-mediated apoptosis [95]. Following the buildup of misfolded proteins inside the ER lumen, large amounts of Ca^2+^ leak into the mitochondria through MAMs, which further enhances the production of ROS by the mitochondrial electron transport chain, owing to the capacity of Ca^2+^ to diffuse to the inner membrane of mitochondria and block complex III eventually causing leaks of electrons and production of free radicals [96,97]. Excessive Ca^2+^ inside the mitochondria leads to further ROS production contributing, thereby, to endothelial dysfunction [61]. Loss of Ca^2+^ homeostasis in the ER can, in turn, stimulate further ER stress and oxidative stress, in a vicious circle, maintaining, therefore, endothelial dysfunction [34].

Recently, Wu et al. [98] demonstrated the role of ER redox homeostasis in homocysteine-mediated endothelial dysfunction. Homocysteine induced the expression of ERO-1α, a major contributor to ER over-oxidation as discussed above, in addition to overexpression of pro-inflammatory molecules (VCAM-1 and ICAM-1) both in vitro in HUVECs and in vivo in thoracic aortas from mice treated with homocysteine (acutely injected or chronically treated with homocysteine). The overexpression of ERO-1α caused by homocysteine was associated with oxidative stress in HUVECs. Homocysteine was found to stimulate mRNA transcription of *ERO-1α* by enhancing the interaction of hypoxia-inducible factor (HIF)-1α with the promoter region of *ERO-1α*. Furthermore, homocysteine allosterically activated ERO-1α by reducing the redox state inside the ER through increasing the ratio of GSH/oxidized glutathione(GSSG) and reducing the expression of PDI. Altogether, the enhanced expression and activity of ERO-1α lead to excess H_2_O_2_, ER stress, inflammation, and, eventually, endothelial cell dysfunction [98]. 

Strategies to alleviate oxidative stress were shown to improve ER stress and endothelial dysfunction in vitro and in vivo. Suganya et al. [99] administered quercetin, a natural anti-oxidant polyphenolic compound, to diabetic STZ-injected rats. STZ-treated rats had increased immunoreactivity to CHOP and ET-1, a potent vasoconstrictor used as a marker of endothelial dysfunction, and decreased expression of the proangiogenic factor VEGF and its receptor (VEGF-R2), in pancreatic tissues, while animals treated with quercetin were protected [99]. Previously, it was shown that the induction of heme oxygenase (HO)-1, a cytoprotective anti-oxidant enzyme, using cobalt-protoporphyrin (CoPP), prevented ER stress-mediated oxidative stress in HUVECs and promoted cell survival and improved angiogenic capacity [58]. Choy et al. [100] recently reported that the co-treatment of mice injected with tunicamycin for two weeks with paeonol, a natural anti-oxidant extracted from the root bark of *Paeonia suffruticosa*, improved ER stress-mediated ROS production and NO bioavailability in tunicamycin-injected mice. The beneficial actions of paeonol were similar to those observed when tunicamycin-treated mice were injected with TUDCA or anti-oxidant, tempol, further supporting a key role for the crosstalk between ER stress and oxidative stress in endothelial dysfunction [100]. 

### 5.3. ER Stress-Mediated Cellular Inflammation And Apoptosis

**Figure 2:**
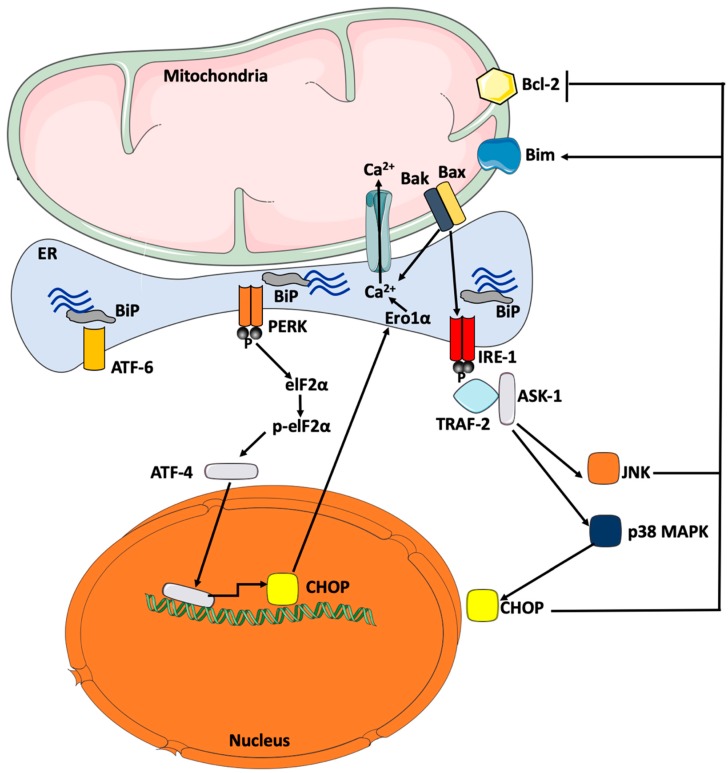
ER stress-induced apoptotic signaling pathways. Tumor necrosis factor-α receptor associated factor (TRAF-2) and apoptosis signal-regulating kinase (ASK)-1 are recruited by IRE-1α triggering the activation of JNK and p38 mitogen-activated protein kinase (MAPK). Activated JNK transfers to the mitochondrial membrane where it concomitantly activates Bim and inhibits Bcl-2. Active p38 MAPK will phosphorylate and, hence, activate CHOP, which in turn, stimulates the transcription of genes involved in apoptosis, such as ER oxidoreductase (ERO)-1α. Bax and Bak also interact and, hence, activate IRE-1α leading to depletion of Ca^2+^ ER stores. Via its kinase activity, PERK phosphorylates the elongation factor, elF-2α, and, hence, reduces general protein translation while allowing selective translation of specific genes ding JNK and NF-κB, which may activate pro-inflammatory and apoptotic signals, such as CHOP.

When UPR response is unable to clear the protein load inside the ER lumen, both the intrinsic and extrinsic signaling pathways of apoptosis will be stimulated. Several key cellular mechanisms involved in cell death response can be triggered by ER stress: I) PERK/eIF-2α-mediated induction of pro-apoptotic transcription factor CHOP; II) IRE-1α-mediated activation of TRAF2, which stimulates the ASK-1/JNK signaling cascade, and III) Bax- and Bcl2-induced Ca^2+^ depletion from ER stores. CHOP has been identified as a major effector for ER stress-mediated apoptosis [101]. Transcription targets for CHOP include growth arrest death domain 34 (GADD34), DR5 (TRAIL Receptor-2), a cell death receptor that activates caspases, and ERO-1α, which hyperoxidizes the ER, thus, exacerbating the stress condition to an unresolvable state promoting cell death. ERO-1α by activating inositol triphosphate receptor (IP3R) may cause excess shuttle of Ca^2+^ from ER stores into the mitochondrial space, which may result in apoptosis [102]. The activation of GADD34 phosphatase by CHOP will mediate the dephosphorylation of elF-2α leading, therefore, to the reactivation of protein translation process [103]. The reactivation of protein translation will, thus, contribute further to the buildup of misfolded and unfolded proteins inside the ER lumen, while selectively and specifically allowing the translation of mRNAs encoding pro-apoptotic molecules. CHOP is also capable of inducing cell death through direct inhibition of anti-apoptotic molecule, Bcl-2 [104] and overexpression of pro-apoptotic molecule, Bim [48], thus, allowing the mitochondrial release of cytochrome c into the cytoplasm triggering, therefore, intrinsic apoptotic reaction cascade. Other ER stress-activated apoptotic mechanisms may include the intrinsic kinase function of IRE-1α which can phosphorylate and, hence, activate ASK-1, p38 MAPK, and JNK; p38 MAPK is also able to activate CHOP via the phosphorylation of its transactivation domain [105]. Furthermore, both p38 MAPK and JNK are known to phosphorylate and, hence, activate Bim and inhibit Bcl-2 with a net result promoting apoptosis [106] (Figure 2).

**Figure 3:**
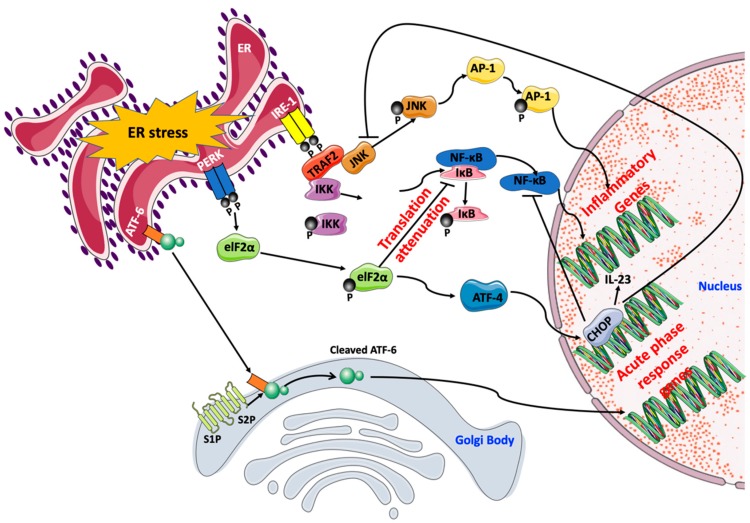
Activation of NF-κB by PERK and ATF-6. The activation of the PERK/eIF-2α axis leads to a severe reduction in protein translation, which decreases the expression levels of IκB protein. Subsequently, the ratio of NF-κB to IκB increases resulting in NF-κB activation. This is associated with a preferential increase in ATF-4 translation and synthesis. ATF-4 induces CHOP, which in turn, causes the induction of the pro-inflammatory cytokine interleukin (IL)-23. The activation of CHOP can also negatively regulate the activity of NF-κB and JNK, thus, affecting the activity of AP-1 downstream. The ER stress effector IRE-1α, activates TRAF-2. This activation complex can then activate IκB kinase (IKK), which in turn, causes IκB degradation and allows free NF-κB to stimulate mRNA expression of inflammatory genes. IRE-1α/TRAF-2 activates JNK, which consequently phosphorylates and, hence, activates the transcription factor AP-1. ATF-6 can induce ER stress-mediated inflammation by leaving the ER and translocating to the Golgi complex to undergo proteolytic cleavage and activation. Active ATF-6 fragments are able to transcribe APR-associated genes, such as those encoding the acute-phase proteins (APPs).

Cellular inflammatory responses activated by ER stress take part in the stress response triggered by metabolic perturbations induced in obesity and diabetes, and whether this inflammation aids in the attenuation of ER stress or aid in its progression depends solely on the ER stress severity and the resistance of the UPR response to be resolved. Several reports indicated that UPR activation is related to the release of multiple pro-inflammatory factors [107]. All three UPR effectors were shown to transcriptionally regulate pro-inflammatory signals with IRE-1α being the most prominent of the three. This regulation is mainly achieved through the action of transcription factors (e.g., NF-κB and activator protein-1 (AP-1)) [108] (Figure 3). 

NF-κB is a central mediator of pro-inflammatory signals. NF-κB family is composed of five members of proteins; p65/RelA, RelB, c-Rel, NF-κB1 (p105/p50), and NF-κB2 (p100/p52). These protein members can either homo- or hetero-dimerize between each other to form active transcription complexes. In non-activated cells, NF-κB dimers localize in the cytosol where they interact with inhibitor of κB (IκB). The stimulation of NF-κB is allowed by proteasome-mediated degradation of IκB facilitating, thus, the translocation of dimers to the nucleus [109]. NF-κB is activated by all three UPR branches via different routes. The activation of NF-κB stimulates the expression of key pro-inflammatory cytokines and effector enzymes of the immune response, such as cyclooxygenase (COX)-2, IL-6, and TNF-α [110,111]. IRE-1α stimulates NF-κB through the degradation of IκB [112]. PERK stimulates NF-κB by blocking protein synthesis of IκB following the phosphorylation of eIF-2α, which frees NF-κB up, thus, allowing its translocation to the nucleus [111]. IRE-1α also activates AP-1, another transcription factor controlling inflammatory gene program activation, via the TRAF2/JNK axis. Genes transcriptionally regulated by AP-1 include for instance TNF-α, keratinocyte growth factor (KGF), IL-8, and some cytokine receptors [113]. Furthermore, UPR can activate a complex inflammatory response referred to as the acute-phase response (APR), which depends on a fine balance mediated by the qualitative nature of intercellular paracrine and/or autocrine signaling [108]. ATF-6 is responsible, in most cells, for activating the cascade of events switching on APR [114] (Figure 3).

ER stress-mediated endothelial apoptosis contributes to the impairment of endothelial functions. HUVECs overexpressing ATF-6 exhibited aggravated ER stress compared to control cells. ATF-6 overexpression enhanced apoptosis in HUVECs through the activation of caspase 3, 9, and 12. In addition, ATF-6 overexpression increased the levels of CHOP and Bax, while simultaneously reduced the levels of Bcl-2. To further contribute to the apoptosis inflicted, the overexpression of ATF-6 upregulated the phosphorylation of JNK (increased p-JNK/JNK ratio) and enhanced the expression levels NF-κB [115]. Previously, Maamoun et al. [34] observed that the exposure of HUVECs to intermittent high glucose stimulated ER stress, reduced NO bioavailability and weakened the capacity of cells to form tube-like structures on a matrigel matrix through a molecular mechanism involving ER stress-mediated apoptosis. These effects were prevented in the presence of PBA and when cells were treated with a chemical inducer of HO-1, an important inducible anti-oxidant and anti-inflammatory enzyme [34], further highlighting the contribution of ER stress-mediated oxidative stress and apoptosis in endothelial cell dysfunction [58]. Bhatta et al. [116] investigated ER stress activation in bone marrow-derived early outgrowth cells (EOCs) treated with high glucose and EOCs from *db/db* diabetic mice and found a significant elevation of ER stress markers compared to controls. In addition, angiogenesis was impaired in EOCs treated with high glucose as shown by the reduction in colony formation, increased apoptosis and decreased migration; all of which were alleviated by pre-treatment of cells with PBA [116].

## 6. Clinical Utility of Targeting ER Stress

Tremendous effort was spent in recent years to develop new small molecules to improve ER homeostasis and reduce ER stress. Most notably, chemical chaperones, PBA and TUDCA, which are both approved by Food and Drug Administration (FDA), were extensively used in the literature to enhance ER protein folding capacity both in vitro and in vivo and were found to be effective in improving glucose metabolism, insulin response, endothelial dysfunction, and cardiovascular function in obesity and diabetes [117]. 

Several modulators targeting the different branches of UPR are now available [117]. Some small molecules were reported for their ability to stimulate the expression of BiP in animals. For instance, valproic acid, a drug approved for the treatment of epilepsy and bipolar disorder, was reported to protect epithelial cells of the retina from apoptosis in a mouse model of ischemia/reperfusion by enhancing the expression of BiP and concomitantly reducing CHOP expression and activation of capsapse 12 [118]. Some FDA-approved anti-hypertensive drugs were also found to protect against ER stress. Guanabenz, an agonist at α2-adrenergic receptors, was found to prolong the activation of eIF-2α by preventing its dephosphorylation via selective inhibition of phosphatase GADD34 [119]. Telmisartan, an angII type-1 receptor blocker, was reported to block apoptotic response mediated by the IRE-1α/TRAF2/caspase 12 axis [120]. Olmesartan is another angII type-1 receptor antagonist that was reported to improve ER stress, oxidative stress, and cellular inflammation. The treatment of STZ-injected diabetic rats with olmesartan prevented ER stress-mediated apoptosis in the kidneys via a mechanism involving the interaction between angII type-1 receptor and CHOP/JNK/caspase 12 axis [121]. Small molecule inhibitors targeting ER membrane-bound effectors of UPR are also available [117]. For instance, kinase inhibiting RNase attenuator 6 (KIRA6) is a potent, competitive, reversible, and selective inhibitor of the kinase activity IRE-1α that was shown to prevent the dimerization and, hence, activation of IRE-1α and to protect cells from cell death under ER stress conditions. The treatment of Akita diabetic mice with KIRA6 reduced ER stress-mediated apoptosis of β-cell islets of Langerhans [122].

Several studies have also reported beneficial actions of some plant-derived compounds on ER stress [117]. Oleanolic acid, a natural compound found in certain plants, was shown to reduce both oxidative stress and ER stress in type-2 diabetic rats and to stimulate the expression of nuclear factor-like 2 (Nrf2) and its target anti-oxidant genes, thereby, contributing to reducing oxidative stress, suggesting a protective role for oleanolic acid against ER stress-mediated complications associated with diabetes [123]. Quercetin, an anti-oxidant flavonoid found in many plants, is another example of natural products found to reduce the activation of ER stress. Quercetin was found to blunt protein expression of BiP in LS180 cells treated with thapsigargin to stimulate ER stress, in addition to the inactivation of UPR sensors, IRE-1α and PERK [124]. Furthermore, quercetin, was reported to reduce the activation of ER stress in HUVECs treated with tunicamycin [125]. 

## 7. Conclusions

Much evidence from both animal and human studies support a key role for the activation of ER stress in the onset and progression of atherosclerosis and other cardiovascular alterations associated with obesity and diabetes. ER stress response is now recognized as a converging molecular link which connects insulin resistance, lipid metabolism disturbances, cell death, and oxidative stress to endothelial dysfunction. Therefore, targeting UPR response pharmacologically represents a promising therapeutic strategy to manage and prevent the development of atherosclerosis in obesity and diabetes.
